# Excision of Clavicle for Oseto Sarcoma

**Published:** 1869-11

**Authors:** N. Senn

**Affiliations:** Ashford, Wis.


ARTICLE XLI.

### EXCISION OF CLAVICLE FOR OSETO SARCOMA.

BY N. SENN, M.D., Ashford, Wis.

Joseph Ammerling, aged 13, a Bohemian boy, applied to me for treatment of a swelling on the left collar-bone, June last.

No predisposition to hereditary disease existed in his family. He always enjoyed good health, until, during the early part of last winter, he noticed a small lump on the anterior surface, at the junction of the middle with the sternal third of the left clavicle. It produced but slight inconvenience, and was only slightly tender on pressure, until the month of May; at that time, it rapidly increased in size; at the same time, it became more painful, especially on moving the arm of the corresponding side, so that when I first saw him, he was unable to use it to any extent.

He then presented a somewhat anæmic appearance; but general nutrition did not appear much impaired. The tumor had attained the size of an ordinary orange, with a broad base, imparting a semi-elastic but firm feel to the touch; taking hold of the clavicle at the distal side of the tumor, it could be moved freely, without affecting the sternal end, producing, at the same time, a grating sound; showing conclusively that there existed a solution of its continuity in the interior of the tumor; there was considerable tenderness on pressure; the skin presented its normal appearance.

As an operation offered the only chances for a recovery, I called in consultation my friend Dr. Marsden. His opinion agreeing with mine, in regard to the diagnosis and treatment of the case, after having obtained the consent of the parents, we operated, June 4th, 1869.

Besides Dr. Marsden, I was assisted by my student, Mr. Jones. After the patient was fully under the influence of chloroform, I made an incision over the centre of the tumor, parallel with the clavicle, extending from near the acromion process to the sterno clavicular articulation. Dividing the skin, superficial fascia, and platysma myoides, the tumor now became apparent beneath the superficial layer of the deep cervical fascia, and the muscles in that region; the former was carefully divided with the knife, from the rest of the structures surrounding the tumor, it was separated with the handle of the scalpel and the fingers—this part of the operation was a tedious one, as the attachments were firm and extensive—the anterior and superior sterno-clavicular ligaments were divided with the knife, then, by inserting an elevator between the articular ends, the posterior attachments were torn through; the tumor was now elevated from its bed, and the clavicle divided with a small metacarpal saw, about an inch and a-half from its distal end. During the operation, the hemorrhage from the small arteries was controlled by pressure with the end of the fingers of the assistants. No ligatures were required. After all venous oozing had ceased, the cavity was washed out with a watery solution of carbolic acid (20–1), and the edges of the wound accurately approximated with nine silk sutures, and supported by long strips of adhesive plaster; a compress, saturated with the same solution, and kept in place with a figure 8 bandage, finished the dressing. The arm was placed in the same position and supported with adhesive plaster, as in fractures of the clavicle. The patient was directed to be kept quiet and placed upon nutritious fluid nourishment.

He slept well during the first night, with but a small amount of anodynes. The day following, he had a little fever, but hardly any pain, and the appetite remained good. On the third day, he insisted on getting out of bed and walking around, as he felt better than before the operation. The first dressing and the sutures were now removed. About two-thirds of the entire wound had united, leaving a wound at each extremity of the incision, healthy in appearance; they were dressed daily with a solution of carb. acid in linseed oil (1–6). The granulations, becoming spongy, were touched every third day with the solid nitrate of silver. The patient improved rapidly, and in about three weeks the wound had entirely healed. There was hardly any suppuration during the whole course of treatment. Along the line of incision there is now a hard ridge of cicatricial tissue, which answers very well for the clavicle, as the patient can move the arm in any direction, and work with it as well as with the opposite one. Except a slight falling forward of that shoulder, and the large cicatrix, nothing would indicate the absence of the clavicle.

The interesting facts about this case are:—1. The slight amount of constitutional disturbance that followed such a severe operation. 2. The almost unimpaired use of the arm, after the removal of almost the entire clavicle. The tumor is oblong and somewhat flat, and measures 6′ in circumference, 3′ in length, and 2½′ in thickness. It is covered by the distended periosteum; its interior is filled with a red, fleshy, granular mass, and softened and destroyed bone, peculiar to all osteo sarcomatous growths. In the centre, the whole thickness of the bone was destroyed. No microscopic examination was made.

---

M. L. CAILLETED demonstrated by numerous experiments that pressure retards all chemical decompositions in proportion to its degree. A plate of zinc lost, under ordinary atmospheric pressure, immersed into muriatic acid, 10.0 parts; under a pressure of 60 atmospheres, 4.7 parts; and, at 120 atmospheres, 0.1 parts, in the same period of time. The boiling point of all liquids is affected similarly, and CAILLETED concludes that all chemical processes are in immediate dependance upon the surrounding mechanical circumstances.—*Journn. Phar. et Chimie.*—*Chicago Pharmacist.*

